# Disease and National Efficiency

**Published:** 1907-11-02

**Authors:** 


### The Hospital

A Journal of

The Medical Sciences and Hospital Administration.

New Series. No. 32, Vol. II. [No. 1104, Vol. XLIII.] SATURDAY, NOVEMBER 2, 1907.

### Disease and National Efficiency.

The study of medical history, though often urged by distinguished voices, cannot be said to have made a very successful appeal either to members of the medical profession or to others outside the professional ranks. The comment is often made that such a study has no "practical" value, and though this is far too sweeping a statement it is not without a measure of justification. But whatever may be said of medical history in its biographical aspects and in its display of the gradual development of opinion and practice, it will at once be recognised that one division of the subject—namely, the study of the history of disease—may be an undertaking of great value and importance. Such a study must, among other results, help to throw light on the conditions under which various diseases have arisen, and on the influences which have determined their distribution. It must thus tend to give a more exact appreciation of the modifying action of such agencies as climate, geography and race on the incidence and manifestation of disease. But it may do something more than this. For, while not ceasing to regard diseases as disturbances conditioned by various circumstances, historical pathology may also turn the subject the other way round, and may proceed to inquire what part disease has played as an influence in the general history of the world. That part, it is certain, must have been a very considerable one, and the complete story of it is probably far beyond reach. But something may be known, and from this definite results may be expected. One of these may be a certain amount of practical guidance for the present and future. Another, and not less important gain may be a quickening of the conscience of governments and of peoples in regard to the relation between the scientific advance of medicine and national welfare and success. For while some progress in this direction may be admitted, it still remains true, as stated a few days ago in a leading article in the *Times*, that the fight against disease, whether in England or abroad, is constantly hindered by "the ignorant contempt of officials and of politicians for the conclusions of science."

The influence of disease on the development and fate of nations has hitherto attracted but little attention. That influence must have been especially active in the case of disease having an endemic character, and bringing under sway a considerable proportion of the inhabitants of an affected district. Particularly must this be true where the disease attacks members of the community in early life. In this way such a degree of prejudice to national efficiency may so readily have been introduced as to handicap fatally the affected people in their struggles with their competitors. And thus the secret of the downfall of nations may in certain instances be found in the endemic disease from which their peoples suffered. This general proposition has recently been applied in a particular instance by Mr. W. H. S. Jones*, of the Perse School, Cambridge, in a scholarly and highly interesting essay. In the course of his historical studies Mr. Jones was led to inquire into the change which manifested itself in the Greek national character during the fourth century before Christ. The explanations usually advanced he judged to be insufficient, and in seeking about for some further cause it occurred to him that disease in the shape of malaria might, at least in part, be responsible. Hence, he set himself to discover whether malaria existed in Greece at the date in question; whether the disease, if it existed, was widely prevalent; and what evidence could be obtained of the effect it produced on character. In his essay Mr. Jones comes to very definite conclusions on these points, and even to those who feel that the case is not quite so clear as the writer seems to think, the argument will prove a very interesting experience. Mr. Jones has also applied his analytical and historical method to the circumstances which attended the downfall of the Roman Empire, and here again he sees reason to conclude that national character suffered under the influence of malaria, and that such deterioration contributed to the general undoing. Whether all his propositions are sound or not, he certainly establishes a presumptive case, and he opens up a line of research which may well be pursued with great advantage.

The essay to which allusion is here made is, however, not content merely to chronicle a series of historical facts and conclusions. It proposes to draw from history lessons of practical significance for the present day, and important more particularly for an Empire which comprises possessions in all latitudes. Here he is welcomed by Professor Ronald Ross, who, without lending an unqualified endorsement to the main thesis of the essay, recognises that the study of malaria in ancient times, and the suggestion of national misfortunes as possibly due to the disease, are agencies tending to arouse active effort towards the suppression of malaria at the present day. At the same time Major Ross quotes one instance in which the disastrous consequences of malaria are strikingly illustrated. This is provided by the experience of Mauritius, where the disease was introduced in 1886, and has caused "infinite injury" ever since. It is a welcome thought that by medical science malaria has been brought into a position in which its complete suppression can be confidently contemplated. The realisation of this possibility rests with an educated public opinion, and as Mr. Jones's essay will help to this end we venture to give it a cordial welcome.

*"Malaria: A Neglected Factor in the History of Greece and Rome." By W. H. S. Jones, M.A. With an introduction by Major R. Ross, F.R.S., C.B., and a concluding chapter by G. G. Ellett, M.B. Cambridge: Macmillan and Bowes, 1907.

